# Screening and Identifying Antioxidative Components in *Ginkgo biloba* Pollen by DPPH-HPLC-PAD Coupled with HPLC-ESI-MS^2^

**DOI:** 10.1371/journal.pone.0170141

**Published:** 2017-01-17

**Authors:** Jiying Qiu, Xiangyan Chen, A. I. Netrusov, Qingxin Zhou, Danyang Guo, Xiaoyong Liu, Hailun He, Xue Xin, Yifen Wang, Leilei Chen

**Affiliations:** 1 Institute of Agro-Food Science and Technology & Shandong Provincial Key Laboratory of Agro-Products Processing Technology, Shandong Academy of Agricultural Sciences, Jinan, China; 2 Biological Faculty, M.V. Lomonosov Moscow State University, 1/12 Lenin’s Hills, Moscow, Russia; 3 School of Life Science, State Key Laboratory of Medical Genetics, Central South University, Changsha, China; Institute of Medical Research and Medicinal Plant Studies, CAMEROON

## Abstract

The *Ginkgo biloba* is one of ancient trees that exists from billions of years ago, its leaf and nut are used as herbs and foods in China, while so far its pollen does not have any application except pollination. In order to evaluate the antioxidant activity of *Ginkgo biloba* pollen, and rapidly screen its antioxidative components, the 1,1-diphenyl-2-picrylhydrazyl (DPPH) scavenging ability, total flavonoid, total phenol, and proanthocyanidin of *Ginkgo biloba* pollen were determined and compared with those of *Ginkgo biloba* leaf and nut, and the off-line DPPH-HPLC-PAD and HPLC-ESI-MS^2^ were applied for screening and identifying the antioxidant flavonoids in *Ginkgo biloba* pollen. The results showed that the DPPH scavenging ability of *Ginkgo biloba* pollen was much higher than *Ginkgo biloba* nut, but lower than *Ginkgo biloba* leaf, while the total content of flavonoid in *Ginkgo biloba* pollen was approximately 4.37 times higher than in *Ginkgo biloba* leaf. Further studies found that the major flavonol aglycone in *Ginkgo biloba* pollen was kaempferol, which accounted for 96.71% of the total aglycones (includes quercetin, kaempferol and isorhamnetin), and the main flavonoid components in *Ginkgo biloba* pollen were flavonoid glycosides. Finally, ten antioxidant peaks were screened and identified to be flavonoids (including kaempferol and nine flavonoid glycosides), so flavonoids were likely to be the main antioxidant components in GP, and among them, three novel kaempferol glycosides (peaks 1, 2, and 3) were found in *Ginkgo biloba* pollen for the first time, which had never been found in *Ginkgo biloba*.

## Introduction

The *Ginkgo biloba* is one of ancient trees that exists from billions of years ago, which is well-known as a “living fossil” in the worldwide area. It was firstly discovered in China, and then rapidly spread into Korea, Japan, Netherlands and other countries and regions [1–2]. As the birthplace of *Ginkgo biloba*, China is one of leading manufacturers of *Ginkgo biloba* medicines [3]. *Ginkgo biloba* leaf (GL) is known for a wide variety of medicinally active chemicals, such as flavonoids (glycosides of kaempferol, quercetin, isorhamnetin, etc.) and terpenoids (ginkgolides and bilobalide) [4–6]. It can be taken internally for the treatment of cerebral and peripheral vascular diseases, ailments associated with ageing (dizziness and ringing in the ears) and short-term memory deterioration, as mentioned in Chinese Pharmacopoeia [7]. However, a few side effects have also been reported, such as intracerbral hemorrhage, gastrointestinal disturbances, headaches, dizziness and allergic skin reactions [8]. Dry *Ginkgo biloba* nut (GN), which contains roughly 60 mg/g sucrose, 680 mg/g starch, 130 mg/g protein and 30 mg/g fat [2], has been widely used as a traditional Chinese medicine to treat asthma, bronchitis, kidney and bladder disorders. The effects, dosages and other useful information of GN can also be found in Chinese Pharmacopoeia [7]. GN also has some toxicity, so it should be eaten according to a certain amount of dosage.

In China, pollens are considered to be safe to eat and have been used as herbs and functional foods for thousands of years. For example, pine pollen and bee pollen can be taken as highly nutritious foods due to their antioxidant and anti-inflammatory activities [9–10]. Similarly, we speculate that *Ginkgo biloba* pollen (GP) is also safe to eat and has the great potential to be made as a functional food. *Ginkgo biloba* pollen includes approximately 236.5 mg/g protein, 15 mg/g V_C_, 13 mg/g V_B1_, 65 mg/g V_B2_, and the contents of total flavonoids and lactones were determined by Wang et al. [11–12], while the studies about its biological activities and the corresponding active substances are not deep enough.

Many oxidative stress-related diseases are caused by the accumulation of free radicals in human bodies. In this regard, flavonoids, proanthocyanidins and other phenols can be used as powerful antioxidants for alleviating the accumulation of free radicals in the body and significantly reducing the chances of having oxidative stress-related diseases for humans [13–15]. DPPH-HPLC-PAD is a simple and rapid approach for screening antioxidant components [16–17], especially for identification and quantitative analysis of antioxidants in Chinese medicinal herbs [18–20]. This method is mainly based upon the reaction between the raw extracts of herbs and 1, 1-diphenyl-2-picrylhydrazyl (DPPH). Due to the reaction, the peak areas of antioxidative compounds in the HPLC chromatogram can be significantly reduced or disappeared [18]. Therefore, antioxidative chemicals can be clearly identified by comparing the chromatograms before and after the DPPH-reaction. HPLC-MS^n^ is an online identification method, it is very useful for the identification of target compounds from impure substances. The active ingredients in plants, such as flavonoids, usually present in a complex matrix of plant extracts, thus it is difficult to isolate enough amounts of highly purified samples for 1H and 13C NMR-spectrometry, 1H-1H-correlated spectroscopy, mass spectrometry, and X-ray crystallography. The hyphenated technique of HPLC-MS^n^ could avoid this drawback. Therefore, HPLC-MS^n^ has been tentatively applied to identify many active ingredients in plants, such as flavonoids in sea buckthorn, phenolic compounds in Bacaba and *Equisetum giganteum*, flavonoids and hydroxycinnamic acids in pak choi varieties [21–25].

Here, in order to study the antioxidant activity and find out the major antioxidative components in *Ginkgo biloba* pollen (GP), the DPPH scavenging ability, the contents of total flavonoid, total phenol, and proanthocyanidin in GP were systematically measured for the first time. Also, a technique that combining off-line DPPH-HPLC-PAD and HPLC-ESI-MS^2^ was used for the first time for antioxidants screening and identification from *Ginkgo biloba* pollen (GP).

## Materials and Methods

### Materials and chemicals

The plant materials: *Ginkgo biloba* pollen (GP) was obtained from the city of Pizhou, Jiangsu Province, China. *Ginkgo biloba* leaf (GL) was purchased from Shandong Bo Kang Traditional Chinese Medicine Co., Ltd (Qingzhou, Shandong, China). Fresh *Ginkgo biloba* fruits were purchased from Qi-Li-Pu Wholesale Market (Jinan, Shandong, China), and *Ginkgo biloba* nut (GN) was stripped out from the fruits.

Reference compounds, including quercetin, kaempferol, and isorhamnetin, were purchased from Shandong Engineering Technology Research Center (Jinan, Shandong, China) with purities of over 98%. 1,1-diphenyl-2-picrylhydrazyl (DPPH), L-Ascorbic acid, and Folin-Ciocalteu’s phenol reagent were purchased from Sigma (St. Louis, MO, USA). Acetonitrile and methanol (HPLC grade) were purchased from Sinopharm (Shanghai, China). Gallic acid, vanillin, catechin, and other chemicals and solvents used in this study were of analytical grade.

### Sample preparation

GP and GL were dried at 55°C for about 3–4 h, and GN was dried at 55°C for 24 h. The dried samples were then crushed into powder of 40 mesh for total flavonoids analysis. Dried powders of GP, GL, and GN (approximately 1.0 g) were immersed in 20 mL of 70% ethanol aqueous solution, then ultrasonicated for 60 min at 25 kHz, 120 W using XO-SM100 Ultrasonic microwave combined reaction system (Nanjing, Jiangsu, China). After cooling to room temperature, each solution was centrifuged at 4,000 rpm and 4°C using CR22GⅢ High-speed refrigerated centrifuge (Hitachi, Tokyo, Japan) to obtain the supernatant. The precipitate was washed with ethanol for three times and centrifuged. All the resulting supernatants were collected and made up to 25 mL. The solutions were analyzed for the measurement of total phenols, proanthocyanidins, DPPH-HPLC-PAD analysis, and HPLC-ESI-MS^2^ analysis. Dried powders of GP, GL, and GN (approximately 1.0 g) were extracted by the ultrasonic method mentioned above, and all the resulting supernatants were concentrated using P-12 Multivapor (BUCHI Labortechnik AG, Flawil, Switzerland), and then dried using FD-1 Freeze dryer (Beijing Boyikang Laboratory Instruments Co., Ltd, Beijing, China). Each dried crude extract was obtained and the extraction yield was calculated by redissolving the dried crude extract in methanol.

### Antioxidant capacity assay by HPLC

The antioxidant capacity assay was carried out by HPLC. Briefly, 500 *μ*L of different concentrations of L-Ascorbic acid (7–70 *μ*g/mL) or samples containing dried crude extracts of GP (2000–7000 *μ*g/mL), GL (1000–5000 *μ*g/mL) and GN (5000–15000 *μ*g/mL) were added to 500 *μ*L of DPPH solution (1.0 mmol/L). The mixture was shaken for a few seconds and left to stand in the dark for 30 min at room temperature.

Then the sample was filtered through a 0.45 *μ*m filter and 10 *μ*L of the sample was injected into the HPLC system for analysis. The blank was prepared by adding 500 *μ*L of methanol to 500 *μ*L of DPPH stock solution (1.0 mmol/L). Chromatographic analysis was carried out by a LC-1200 high performance liquid chromatograph (HPLC) (Agilent, Palo Alto, CA, USA) equipped with an Agilent ZORBAX Eclipse XDB-C18 column (150 mm×4.6 mm i.d., 5*μ*m) and a photodiode array detector (PAD). The mobile phase of (A) 0.2% acetic acid in water and (B) methanol (25:75, v/v) was isocratic eluted at a flow rate of 1.0 mL/min. The DPPH peaks were monitored at 517 nm. The difference in the reduction of DPPH peak area (PA) between the blank and the sample was used for determining the DPPH scavenging activity of the sample according to a reported formula [26].

$$DPPH\;inhibition\;rate\;\left(\%\right)=\left[1\;\text{-}\;PA_{\left(sample\right)}/PA_{\left(blank\right)}\right]\times 100$$

IC_50_ (50% inhibition concentration) values, which represent the concentration of sample required to scavenge 50% of DPPH, were calculated by nonlinear regression analysis. L-Ascorbic acid was used as a radical scavenger reference compound.

### Total phenols analysis

Total phenols were estimated by the slightly modified Folin-Ciocalteu method [27–28]. Briefly, 0.05 mL of each diluted solution of extracts was added to 0.1 mL of 2 mol/L Folin-Ciocalteu’s phenol reagent (Sigma, St. Louis, MO, USA). Then 0.2 mL of sodium carbonate solution (120 g/L) was added in the reagent after 30 s to 8 min and made up to 2.5 mL. The tube was laid for 5 min in a water bath at 50±0.5°C and then put in a cold water bath. The absorbance at 760 nm was measured. 0.05 mL of methanol was used instead of extracts as a control sample. The amounts were expressed in milligrams of gallic acid equivalents (GAE) per gram of dry matter. The standard curve was prepared with aqueous solutions of pure gallic acid.

### Proanthocyanidins analysis

The method is based on the condensation of the vanillin onto the phloroglucinol nucleus catalysed by H_2_SO_4_ [27]. 0.5 mL of each diluted solution of extracts was added to 2.5 mL of freshly prepared solution of vanillin/methanol (30 g/L), and then a 2.5 mL of 30% H_2_SO_4_/methanol (v:v) was added. After reaction in a water bath at 30±0.5°C for 20 min, the absorbance at 500 nm was measured. For a control sample, 0.5 mL of methanol was used instead of extracts. The amounts were expressed in milligrams of catechin per gram of dry matter. The standard curve was prepared with aqueous solutions of pure catechin.

### Total flavonoids analysis

The flavonoids of *Ginkgo biloba* can be reduced by hydrolysis to three major aglycones, namely isorhamnetin, kaempferol and quercetin. The total flavonoid contents were determined by the method reported by Hasler et. al [29] after slight changes. A 1.0 g of material was refluxed with 20 mL of methanol and 5 mL of 25% hydrochloric acid for 60 min. After cooling, the solution was centrifuged at 4,000 rpm and 4°C using High-Speed Refrigerated Centrifuge CR22GⅢ (Hitachi, Tokyo, Japan). The precipitate was washed with methanol for three times and centrifuged. The solutions were collected together and diluted to 50 mL with methanol in a volumetric flask.

Then the solutions were filtered through a 0.45 *μ*m filter. The flavonol aglycones, isorhamnetin, kaempferol and quercetin, were analyzed using an Agilent 1200 LC system mentioned above. The mobile phase containing a mixture of 50% solvent A (0.2% acetic acid in water) and 50% solvent B (methanol). The flow rate was set at 1.0 mL/min, the column temperature was maintained at 35°C, the injection volume was 10 *μ*L and the detection wavelength was performed at 360 nm. The contents of quercetin, kaempferol, and isorhamnetin were determined and the total flavonoids contents were calculated by a formula of *total flavonoids content* = *quercetin* × 2.51 + *kaempferol* × 2.64 + *isorhamnetin* × 2.39 [30].

### DPPH-HPLC-PAD analysis

An off-line DPPH-HPLC-PAD method [26] was applied to screen the main antioxidants from GP. The sample was mixed with 10 mmol/L DPPH at the ratio of 1:1 (v:v) and incubated at room temperature for 30 min. Then the mixture was filtered through a 0.45 *μ*m filter, and injected into the HPLC column. The control sample was prepared by adding methanol instead of DPPH to the sample.

The DPPH-reacted sample and control sample were analyzed using a LC-1200 high performance liquid chromatograph (HPLC) (Agilent, Palo Alto, CA, USA) equipped with an Agilent ZORBAX Eclipse SB-C18 column (250 mm×4.6 mm i.d., 5*µ*m) and a photodiode array detector (PAD). The mobile phase consisted of 0.2% (v/v) acetic acid (A) and acetonitrile (B) using a gradient program of 12–21% B within 0–30 min, 21–32% B within 30–40 min, 32–55% B within 40–50 min, 55–65% B within 50–80 min and 65% B within 80–85 min. The flow rate was 1.0 mL/min, the column temperature was 30°C, the injection volume was 10 *μ*L and the detection wavelength was 265 nm. The main antioxidants could be screened by comparing the chromatographic profiles of DPPH-reacted sample and control sample.

### HPLC-ESI-MS^2^ analysis

Q-TOF LC/MS^2^ has been widely used to tentatively identify the screened antioxidants by analyzing their molecular weights. To this purpose, an Agilent Q-TOF LC/MS equipped with an electrospray interface was performed (Agilent 6520, Palo Alto, CA, USA). The HPLC-ESI-MS^2^ analysis was performed under the same HPLC condition for DPPH-HPLC-PAD analysis. Q-TOF/MS and MS^2^ analyses were carried out using both positive and negative modes, with a scan range from *m/z* 50 to 1000 Da. The MS^2^ spectrums were acquired under an automatic MS^2^ acquisition mode. Operating parameters of MS and MS^2^ were listed as follows: drying gas (N_2_) flow rate, 10.0 L/min; drying gas temperature, 350°C; Nebulizer, 35 psi; capillary voltage, 3500 V; fragmentor 175 V; skimmer voltage, 65 V; and octopole radio frequency, 750 V.

### Statistical analysis

The DPPH scavenging ability, total flavonoid, total phenol, and proanthocyanidin were determined in triplicate. Data were expressed as mean ± standard deviation (SD). Statistical differences between samples were identified using one-way ANOVA followed by the Tukey post-test and significant difference was determined between mean values of the samples using comparison test at the level of *p*<0.01.

## Results and Discussion

### Comparative study of antioxidant capacities and potential antioxidant components

In order to investigate the antioxidant capacity and antioxidant components of GP, the DPPH scavenging abilities of GP, GL and GN were evaluated, and the potential antioxidant components of GP, GL and GN, namely total phenols, proanthocyanidins and total flavonoids, were determined. By measuring DPPH scavenging activities, the IC_50_ values of GP, GL, and GN before and after extraction were determined. The IC_50_ values of GP, GL and GN powders before extraction were calculated to be 12.80±0.11, 5.42±0.02, and 50.37±0.36 mg/mL, respectively. After extraction, the IC_50_ values of the crude extracts were 4.02±0.03 (GP), 1.20±0.01 (GL), and 8.33±0.06 mg/mL (GN), respectively. It showed that the antioxidant capacity of GP was lower than that of GL (*p*<0.01), but much higher than that of GN (*p*<0.01). However, the antioxidant capacities of GP, GL and GN were all lower than that of L-ascorbic acid as a control with an IC_50_ value of 39.90±0.04 *μ*g/mL (*p*<0.01). The compositions of potential antioxidant components were shown in Table 1. The contents of total phenols, proanthocyanidins, and total flavonoids in GP, GL, and GN were significantly different (*p*<0.01). The antioxidant components in GP were dominated by flavonoids, and it also contained a small amount of phenols and proanthocyanidins. The total flavonoid content in GP was approximately 4.37 times higher than that in GL (*p*<0.01) and was extremely higher than that in GN (*p*<0.01), and the total phenol and proanthocyanidin in GP were all lower than that in GL (*p*<0.01). Therefore, flavonoids were likely to be the main antioxidant components in GP.

**Table 1:** Total phenols, proanthocyanidins, and total flavonoids of *Ginkgo biloba* pollen (GP), *Ginkgo biloba* leaf (GL) and *Ginkgo biloba* nut (GN) (mg/g)^a^.

Samples	Total phenols	Proanthocyanidins	Total flavonoids
**GP**	1.0018±0.0020	0.8400±0.0016	16.3149±0.2947
**GL**	1.2022±0.0023	2.8825±0.0035	3.7328±0.0042
**GN**	0.2050±0.0004	ND^b^	0.0112±0.0001

^a^The amounts were all expressed as mean±SD in milligrams per gram of dry matter before extraction, and the contents of total phenols, proanthocyanidins, and total flavonoids in GP, GL, and GN were significantly different (*p*<0.01).

^b^“ND” means “not detected”.

In order to study the differences of the flavonoids among GP, GL and GN, the contents of flavonol aglycones (quercetin, kaempferol, and isorhamnetin) after acid hydrolysis were also analyzed (Table 2). The contents of quercetin, kaempferol, and isorhamnetin in GP, GL, and GN were significantly different (*p*<0.01). Their contents of quercetin, kaempferol, and isorhamnetin in GL accounted for 41.87, 50.36, and 7.77% respectively, while the major flavonol aglycone in GP was kaempferol, which accounted for 96.71% of the three major aglycones. Thus, it can be speculated that the species and the contents of flavonoids in GP were probably very different from those in GL.

**Table 2:** Three flavonol aglycones of *Ginkgo biloba* pollen (GP), *Ginkgo biloba* leaf (GL) and *Ginkgo biloba* nut (GN) (mg/g)^a^.

Samples	Quercetin	Kaempferol	Isorhamnetin
**GP**	0.1467±0.0001	5.9856±0.0001	0.0605±0.0001
**GL**	0.6090±0.0007	0.7326±0.0009	0.1130±0.0001
**GN**	0.0045±0.0001	ND^b^	ND^b^

^a^The contents of quercetin, kaempferol, and isorhamnetin were determined after acid hydrolysis, and were all expressed as mean±SD in milligrams per gram of dry matter before hydrolysis. The contents of quercetin, kaempferol, and isorhamnetin in GP, GL, and GN were significantly different (*p*<0.01).

^b^“ND” means “not detected”.

### Comparative study of flavonoids species

**Fig 1:**
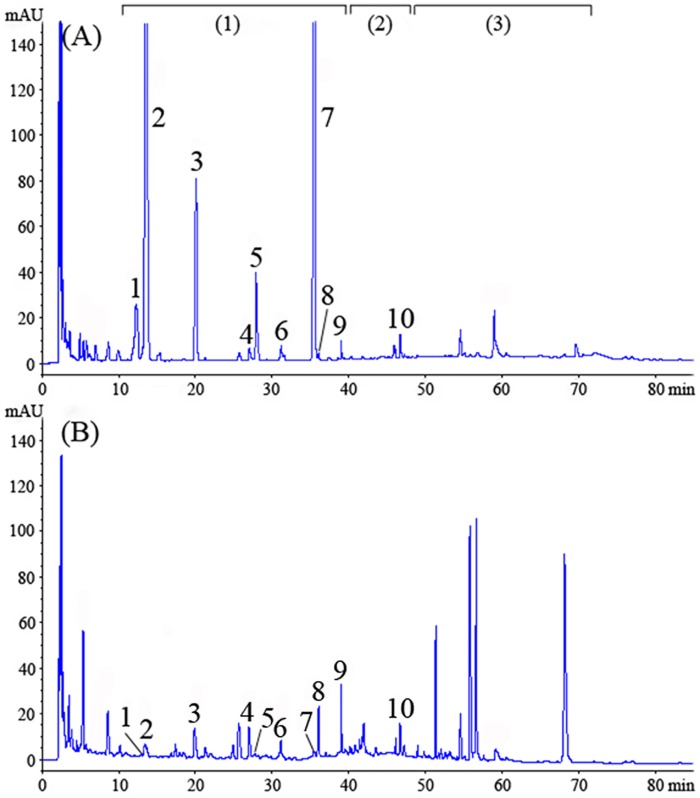
HPLC chromatograms of extracts of *Ginkgo biloba* pollen (GP) and *Ginkgo biloba* leaf (GL). (A) *Ginkgo biloba* pollen (GP), (B) *Ginkgo biloba* leaf (GL); (1) flavonoid glycosides, (2) flavonol aglycones, (3) biflavones; Peaks 1–10: antioxidant peaks screened from *Ginkgo biloba* pollen (GP) by off-line DPPH-HPLC-PAD method (see Fig 2).

The HPLC spectrums of flavonoids in GP and GL were compared after HPLC separation and detection. The same HPLC conditions were used as DPPH-HPLC-PAD analysis. As shown in Fig 1, GP and GL are quite different in the species of flavonoid glycosides and the content of each flavonoid glycoside. Flavonoid glycosides are the main flavonoids in GP, their total peak areas (retention times from 12 to 40 minutes) can be roughly accounted for 95% of that of the total flavonoid (retention times from 12 to 72 minutes), while biflavones (retention times from 50 to 72 minutes) were the main flavonoids in GL. The total peak areas of flavonoid glycosides in dry GP was approximately 9.93 times higher than that in dry GL. Besides, the peak areas of flavonol aglycones and biflavones in GP were extremely less than those in GL. Ding et. al [31] reported that there were flavonoid glycosides, flavonol aglycones, and biflavones in GL. Flavonoid glycosides were the main antioxidant flavonoids in GL, flavonol aglycones were hardly found and thus they did not contribute significantly to the overall activity, although they are probably performed similar or higher activities as other glycosides, and biflavones hardly inhibit radicals. Therefore, antioxidants screening in GP, especially for antioxidant flavonoid glycosides is very meaningful.

### DPPH-HPLC-PAD screening of main antioxidants in *Ginkgo biloba* pollen (GP)

Reaction between an antioxidant and a radical such as DPPH could result the oxidation of antioxidant, which can change the molecular structure of antioxidant. In this regard, the DPPH-HPLC method was developed to screen antioxidant compounds from multi-component materials, such as traditional Chinese medicine and food materials [19,32]. For crude extracts with antioxidant effects, the peak areas of the radical scavenging compounds in the chromatographic profiles would be obviously decreased after reacting with DPPH. In contrast, for those without antioxidant effects, there were almost no changes in their peak areas. Chromatographic conditions mainly referred to literature [31], and the gradient elution conditions were optimized for better separation of flavonoid glycosides in GP. Most of the antioxidant flavonoid glycosides (especially with high absorbance) in GP were well separated after optimization (Fig 1A).

**Fig 2:**
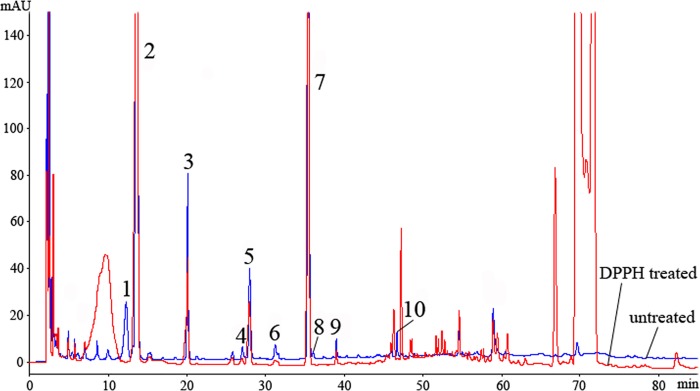
Chromatograms of *Ginkgo biloba* pollen (GP) extracts with UV at 265 nm detection of antioxidant flavonoids based upon DPPH elimination. Ten antioxidant peaks 1 through 10 were labeled in the profile.

The untreated and DPPH-treated GP extracts were analyzed by HPLC and compared. Ten peaks were found to be decreased after spiking with the DPPH solution (see Fig 2), and the peak areas of peaks 1 through 10 before and after reacting with DPPH were compared and the rates of decline after the reaction were calculated by a formula of the rates of decline (%) = (1—PA_2_ / PA_1_) × 100. It was shown in Table 3 that the peak areas of these ten peaks all declined, and even peaks 1 and 8 completely disappeared after the reaction. It indicated that these peaks were all antioxidants present in the GP extract, although their antioxidant capacity cannot be quantified by the rates of decline. On the other hand, some peaks with retention time near 50 and 70 minutes appeared or significantly increased after reacting with DPPH, and they were the residual DPPH and the reaction products [26]. In addition, flavonoid glycosides were mostly responsible for DPPH scavenging of GP, and the contents of the main antioxidant flavonoid glycosides in GP, peaks 2, 3, 5, and 7, were much higher than those in GL (Fig 1).

**Table 3:** The peak areas of peaks 1 through 10 before and after reacting with DPPH and the rates of decline (%).

Peaks	PA_1_^a^	PA_2_^b^	The rates of decline (%)
**1**	852.25	0	100.00
**2**	14904.15	9785.14	34.35
**3**	1264.92	1041.94	17.63
**4**	80.79	61.68	23.65
**5**	627.95	526.72	16.12
**6**	104.37	46.22	55.72
**7**	7934.48	4033.41	49.17
**8**	44.40	0	100.00
**9**	76.65	68.41	10.75
**10**	88.49	13.45	84.80

^a^PA_1_: Peak areas before reaction.

^b^PA_2_: Peak areas after reaction.

### HPLC-ESI-MS^2^ identification

Ten antioxidant compounds screened from GP (see Fig 2) were identified by HPLC-ESI-MS^2^. Their mass informations of maximum absorption of UV, [M+H]^+^, [M-H]^-^, and fragments were shown in Table 4, and their corresponding molecular weights, molecular formulas and identification results were shown in Table 5. According to fragment informations and some references [31,33–35], peaks 1 to 9 were tentatively identified to be flavonoid glycosides, peak 10 was identified to be kaempferol, which was the main flavonol aglycone in GP. Among the identified antioxidant flavonoid glycosides, most of them were kaempferol glycosides, excluding two small peaks, peaks 4 and 8. In detail, the structures of peaks 2, 3, 5, 6, 7, and 10 were identified by fragment information analysis, and further confirmed by comparing with standard substances, peaks 4, 8, and 9 were identified by comparing retention times and fragment informations with the identified peaks of 9, 11 and 12 in *Ginkgo biloba* leaf (GL) as reported by Ding et. al [31], peak 1 was deduced to be a kaempferol glycoside linked with two glucosyl groups by fragment information analysis, however, its structure needs to be further identified by a lack of standard substance and reference. Among these peaks, peaks 1, 2, and 3 were found in *Ginkgo biloba* for the first time.

**Table 4:** λ_max_, [M+H]^+^, and [M-H]^-^ of the ten antioxidant peaks in *Ginkgo biloba* pollen (GP) by HPLC-ESI-MS^2^.

Peaks	λ_max_ (nm)	[M+H]^+^ (*m/z*)	[M-H]^-^ (*m/z*)
**1**	265, 338	611.1615, 287.0547, 85.0275	609.1425, 447.1007, 283.0279, 255.0332
**2**	266, 343	611.1615, 287.0553, 85.0288	609.1425, 446.0904, 283.0271, 255.0319
**3**	265, 318, (336)	595.1658, 287.0552, 85.0294, 71.0505	593.1461, 446.0949, 283.0278, 255.0323
**4**	253, (268), 356	625.1766, 317.0654, 85.0282, 71.0483	623.1564, 315.0544, 300.0296, 271.0257, 243.0318
**5**	265, 348	449.1077, 287.0549, 85.0295	447.0879, 284.0367, 255.0336, 227.0373
**6**	265, (321), 362	449.1077, 287.0541	447.0879, 284.0354, 151.0053, 107.0138
**7**	265, 344	433.1141, 287.057, 85.0307, 71.0513, 57.0354	431.0926, 285.0449, 255.0342, 227.0381
**8**	266, 315	757.1959, 345.0617, 147.0438	755.1761, 609.1646, 300.0313, 271.0281, 178.9990, 151.0043
**9**	268, 314	741.2032, 329.0655, 147.0434, 119.0498	739.1797, 593.1679, 284.0379, 255.0336, 227.0379, 145.0301
**10**	266, (318), 368	287.0552, 165.0176, 153.0183, 121.0284	285.0340

**Table 5:** Identification of the ten antioxidant peaks in *Ginkgo biloba* pollen (GP) by HPLC-ESI-MS^2^.

Peaks	M (*m/z*)	Formula	Tentative identification
**1**	610.15	C_27_H_30_O_16_	3,7-Di-*O-*(*β*-D-glucosyl) kaempferol^a^
**2**	610.15	C_27_H_30_O_16_	3,4’-Di-*O-*(*β*-D-glucosyl) kaempferol
**3**	594.15	C_27_H_30_O_15_	3-*O*-(*β*-D-glucosyl)-7-*O*-(*α*-L-rhamnosyl) kaempferol
**4**	624.16	C_28_H_32_O_16_	3-*O*-[6-*O*-(*α*-L-rhamnosyl)-*β*-D-glucosyl] isorhamnetin
**5**	448.10	C_21_H_20_O_11_	3-*O*-(*β*-D-glucosyl) kaempferol
**6**	448.10	C_21_H_20_O_11_	7-*O*-(*β*-D-glucosyl) kaempferol
**7**	432.10	C_21_H_20_O_10_	3-*O*-(*α*-L-rhamnosyl) kaempferol
**8**	756.18	C_33_H_40_O_20_	3-*O*-{2-*O*-[6-*O*-(*p*-hydroxy-*trans*-cinnamoyl)-*β*-D-glucosyl]-*α*-L-rhamnosyl}quercetin
**9**	740.19	C_33_H_40_O_19_	3-*O*-{2-*O*-[6-*O*-(*p*-hydroxy-*trans*-cinnamoyl)-*β*-D-glucosyl]-*α*-L-rhamnosyl}kaempferol
**10**	286.04	C_15_H_10_O_6_	Kaempferol

^**a**^one isomeric compound of peak 2, likely 3,7-Di-*O-*(*β*-D-glucosyl) kaempferol.

## Conclusions

Flavonoids, phenols, and proanthocyanidins are the major antioxidant components existing in plants. In this article, the antioxidant capacity and the contents of total flavonoid, total phenol, and proanthocyanidin of *Ginkgo biloba* pollen (GP) was qualitatively analyzed. A technique that combining off-line DPPH-HPLC-PAD and HPLC-ESI-MS^2^ was used for the first time for antioxidants screening and identifying from GP. The results showed that flavonoids were very important active compounds present in GP, they possibly can be the main components that generate the antioxidant effect. Moreover, the flavonoids species of GP were found significantly different from GL and GN, kaempferol was the major flavonol aglycone in GP, and flavonoid glycosides were its main flavonoids. Meanwhile, ten antioxidant compounds were screened out and mainly identified as flavonoid glycosides. Among them, three novel kaempferol glycosides (peaks 1, 2, and 3) were discovered for the first time, which had never been found in *Ginkgo biloba* leaf and nut. It seemed that *Ginkgo biloba* pollen (GP) is rich in antioxidant flavonoids, especially water-soluble flavonoid glycosides, we considered that the *Ginkgo biloba* pollen (GP) has greater potential for the market of healthy foods when compared with *Ginkgo biloba* leaf (GL) and *Ginkgo biloba* nut (GN).
